# Changes in flexion-relaxation phenomenon and lumbo-pelvic kinematics following lumbar disc replacement surgery

**DOI:** 10.1186/1743-0003-10-72

**Published:** 2013-07-10

**Authors:** Julie O’Shaughnessy, Jean-François Roy, Martin Descarreaux

**Affiliations:** 1 Département de chiropratique, Université du Québec à Trois-Rivières (UQTR), Trois-Rivières, Québec, Canada; 2 Orthopaedic Surgeon, Centre Hospitalier Universitaire de Québec (CHUQ), Hôpital St-François d’Assise, Québec, Québec, Canada; 3 Département de chiropratique, UQTR, Trois-Rivières, Québec, Canada

**Keywords:** Low back pain, Disc prosthesis, Degenerative disc disease, Flexion-relaxation phenomenon, Lumbar kinematics

## Abstract

**Background:**

A single group prospective study. Disc prostheses are believed to contribute to the restoration of the segmental movement and the preservation of the adjacent segments. The study’s main objective was to determine if changes in neuromuscular patterns assessed using the flexion-relaxation phenomenon (FRP) can be observed following disc replacement surgery.

**Methods:**

Fifteen subjects participated in this study; they were evaluated before and after lumbar disc replacement surgery. Both assessments included ten repetitions of a trunk flexion and extension movement (with and without a load), where the surface electromyography (EMG) and kinematic data were recorded.

**Results:**

Following the disc replacement procedure (17.3 weeks ± 8.4), participants reported a significant reduction in their ODI and FABQ - physical activity scores. Increases in pelvic flexion as well as in erector spinae (ES) muscle activity at L5 in the flexion phase were observed. Following the disc replacement surgery, ES activity at L2 decreased during the quiet standing position.

**Conclusion:**

The results of this study suggest that although improvements in disability scores and fear-avoidance related to physical activities scores were noted after a disc replacement surgery, the lumbar ROM was not modified. Nevertheless, a significant increase in the hip ROM during the flexion-extension task as well as an increase in ES muscle activity in flexion was observed following surgery. The VAS, FABQ I and ODQ scores were positively correlated with change in the muscular activities during the FRP.

## Background

Elective surgery can reduce pain and decrease disability in chronic low back pain (CLBP) patients when conservative therapies are not effective and daily life activities are severely limited [1]. It has been estimated that between 6 and 7.5% of the CLBP patients undergo spinal surgery in the United States [2,3]. The most common elective surgeries for degenerative disc disease (DDD) can be divided into two broad categories: fusion (arthrodesis) and disc replacement (arthroplasty) [4-7]. Arthroplasty has been practiced in Europe since 1980, but only since the turn of the century in the United States [8]. This new procedure is believed to offer some advantages over fusion. Indeed, its anterior approach to removing the symptomatic degenerative disc and replace it by a prosthesis spares the posterior elements and protects the posterior neurological and vascular structures [7,8]. The few comparative studies between fusion and disc replacement showed better outcomes for disc replacement with regard to operative time, blood loss, hospitalization stay and use of narcotics [6,9-12]. Compared to a vertebral fusion, patients with disc prosthesis showed better clinical results measured with the Oswestry Disability Index (ODI), the SF-36 Health Survey Questionnaire (SF-36) and the Visual Analogue Pain Scale (VAS). Patients also showed higher levels of satisfaction following disc replacement surgery [9,10,13]. From a biomechanical standpoint, disc prostheses are believed to contribute to the restoration of segmental movement and the preservation of the adjacent segments. Disc replacement surgery was developed as an alternative to fusion’s long term complications such as the increase of mechanical stress and load on the adjacent segments. These increased loads can lead to post-surgical vertebral instability, early disc degeneration and pain. In fact, symptomatic adjacent segment disease (ASD) is found in 5.2 to 18.5% of the patients who present with CLBP after a fusion and can lead to a second surgical intervention, often with limited results [14,15]. In a systematic literature review on symptomatic ASD (only four articles reported), Harrop et al.[16] estimated that this type of complication was only present in 1% of the patients at 8.7 to 13.2 years following arthroplasty. Besides the clinical improvement and the reduction of the symptomatic ASD, one proposed advantage of the disc prosthesis is to emulate the normal disc biomechanics [17]. Disc replacement might preserve or increase the segmental range of motion (ROM), thus keeping the physiologic lumbar spinal kinematic properties [10,11,18-20].

Spinal stability in a «healthy» lumbar spine (absence of symptoms, degeneration or surgical intervention) involves complex interactions between neuromuscular control and passive tissues [21,22]. In CLBP patients, neuromuscular adaptations are developed to perform daily tasks. These adaptations can be objectively observed and quantified using, for example, a simple flexion-extension task [23]. During a flexion-extension task, healthy subjects (without low back pain) exhibit a reduction in, or a silence of the electromyographic (EMG) signal of the lumbar erector spinae (ES). Myoelectric silence of the superficial ES muscles when approaching full flexion was first observed in 1948 by Allen, and later by Floyd and Silver who called the phenomenon «flexion-relaxation» (FRP) [24-26]. In the presence of low back pain, the EMG silence is absent during full flexion. It is unknown if the continuous contraction of the ES is due to the subjects’ pain, or if the constant muscular activity induces the pain, but studies have showed that this absence of FRP can be observed in healthy subjects submitted to experimental pain [27,28]. This neuromuscular response has been thoroughly investigated and appears to be the result of equilibrium between the gravity-induced flexion moment and the stretched posterior structures, namely the posterior ligaments and the lumbar ES [29,30]. Because FRP reliability (inter- and intra-raters) measured with kinematic and surface EMG is excellent, it is often used as an objective clinical improvement measurement tool [31-35].

Although FRP has been used to monitor clinical improvement in chronic non-specific low back pain, only a few studies about specific low back conditions have been published. One study measured the FRP before and after herniated nucleus pulposus in one subject, and found that the FRP returned to its initial status (absence of the EMG signal in lumbar flexion) when the patient’s symptoms resolved and the lumbar ROM returned to its normal amplitude [36]. One study reported FRP parameters before and after lumbar discectomy surgery in 17 subjects with disc herniation [37]. After four weeks, the EMG recordings showed a continuous contraction of the ES during lumbar flexion, despite the decreased pain level.

Disc replacement surgery represents a unique model to study the neuromuscular adaptation before and after surgery in low back pain patients. Since the procedure is believed to maintain or improve ROM and overall functional capacity, the aim of this study is to compare, in a group of participants with DDD, trunk neuromuscular control and trunk kinematics before and after a disc replacement surgery. It is hypothesized that, together with decreased levels of pain and disability, flexion-relaxation parameters will be normalized following the surgery. This study is the first to observe the neuromechanical adaptations following disc replacement surgery.

## Methods

### Participants

Fifteen participants (ten men and five women) between 32 to 58 years old (mean: 43.5 ± 8.6) were recruited for this study. The participants included in the present study were all scheduled for elective disc replacement surgery for CLBP (from DDD) and were all recruited from the same orthopaedic clinic. The general inclusion/exclusion criteria for arthroplasty were determined by an orthopaedic surgeon (JFR) and are reported in Table 1. A discography analysis was ordered for all patients to confirm the magnetic resonance imaging or computer tomography findings and the origin of the discogenic pain. Participants with a body mass index (BMI) > 30 and a waist circumference > 102.01 centimeters for men and > 88.01 centimeters for women were excluded from the study. This work was approved by both the Université du Québec à Trois-Rivières and the Centre Hospitalier Universitaire de Québec research ethical review boards. All participants gave their written informed consent and their participation in the study had no influence on the surgical protocol and clinical follow-ups.

**Table 1:** Inclusion and exclusion criteria for lumbar arthroplasty

**Inclusion**	**Exclusion**
Adults between 18 and 60 years old (bone maturity).	DDD > 1 symptomatic vertebral level
Degenerative disc disease (DDD) between L1 and S1.	Vertebral end-plates smaller than 34.5 mm in the media-lateral plan and/or 27 mm in the antero-posterior plan.
DDD certified by:	Allergy to :
▪ Low back pain and/or leg pain (radiculopathy) and	▪ Titanium
	▪ Polyethylene
▪ CT scan, MRI, discography, radiology, myelography and/or flexion/extension radiographs with at least one of the following:	▪ Cobalt
	▪ Chrome
	▪ Molybdenum
- Instability ( ≥ 3 mm translation or ≥ 5° degrees);	Past vertebral surgery (thoracic or lumbar) ▪ Fusion
- Loss of disc high > 2 mm	▪ Bilateral spinal or unilateral vertebral decompression where > 50 % of the facet was removed
- Thickness/scar of the annulus pulposus	
- Herniated nucleus pulposus; or	▪ Facet fracture
- Vacuum phenomenon	
Oswestry disability index ≥ 40 (20/50)	Trauma (past or present) to the vertebral end-plates.
VAS ≥ 40/100	Pregnancy
Failure to improve with a trial of nonsurgical management (physical therapy, medications and epidural injection).	

*CT* computed tomography, *MRI* Magnetic resonance imaging, *mm* millimeter.

### Experimental protocol

The experimental sessions (biomechanical laboratory) were conducted before and after the disc replacement surgery (at least three months) and each testing session lasted approximately 90 minutes. Prior to postoperative laboratory assessments, patients were examined by the orthopedic surgeon and a clinical assessment and flexion-extension x-ray were performed. In the absence of surgical complications, and when stability of the prosthesis was confirmed by the surgeon, participants were referred for their second postoperative laboratory assessment.

### Outcome measures

Participants were asked to complete the French versions of the modified Oswestry Low Back Pain Disability Questionnaire (ODQ) and the Fear-Avoidance Beliefs Questionnaire (FABQ), respectively to assess disability and fear-avoidance behaviors related to their CLBP. These questionnaires demonstrate moderate conceptual validity and good reproducibility [38,39]. The clinical pain intensity was assessed using a visual analogue scale (VAS). The VAS has been found superior to the McGill pain questionnaire to detect pain responsiveness in low back pain patients involved in postsurgical rehabilitation [40]. The participants were asked to complete the questionnaires before each laboratory session. Trunk and hamstring flexibility were also measured before and after the surgery with the sit and reach test [41].

### Experimental task and procedures

Participants were asked to perform a trunk flexion-extension task. Verbal instructions, followed by a demonstration and practice trials, were provided before the experiment. During the task, participants stood with arms crossed over their chest with their legs fully extended. The complete cycle of movement was characterized by four different movement phases: (1) standing still (three seconds); (2) trunk flexion to reach a fully-flexed state (five seconds); (3) full flexion (three seconds) and (4) trunk extension to return to the initial upright position (five seconds). A metronome was used to standardize the task’s speed and duration. Ten flexion-extension cycles were completed; five without load and five during which the participants held 10 kg. During this “loaded condition”, the weight was held with hands crossed over their chest. The two different conditions were performed in blocks of five trials and the order in which they were performed was randomized across participants.

### Instrumentation

Surface EMG data were collected using bipolar disposable surface Ag-AgCl electrodes applied bilaterally over the ES muscles at L2 and L5 level, approximately 3 cm from the mid-line of the spine (electrodes were applied in-line with muscle fiber orientation) [42]. A ground electrode was placed on the left anterior superior iliac spine. Skin impedance was reduced by shaving body hair, gently abrading the skin with fine-grade sandpaper (Red Dot Trace Prep, 3M, St. Paul, MN, USA), and wiping the skin with alcohol swabs. The EMG activity was recorded with a Bortec biomedical acquisition system (Model AMT-8, common mode rejection ratio of 115 dB at 60 Hz, input impedance of 10 GΩ) and sampled at 1000 Hz with a 12-bit A/D converter (PCI 6024E, National Instruments, Austin, TX). The EMG data were digitally filtered by a 10- to 450-Hz band-pass, dual-pass zero-lag, fourth-order Butterworth filter. The data were collected using LabView (National Instruments) and analyzed using MatLab (Mathworks, Natick, MA).

Kinematic data were collected by a motion analysis system (Optotrak Certus, Northern Digital, Waterloo, ON, Canada). Light-emitting diodes (LED) were positioned on the right side of each participant over eight different anatomic landmarks: (1) lateral malleolus; (2) head of the fibula; (3) lateral condyle of the femur; (4) greater trochanter; (5) anterior superior iliac spine (ASIS); (6) posterior superior iliac spine (PSIS); (7) L1 and (8) T11. Kinematic data were collected at 100 Hz and low-pass filtered by a dual-pass zero-lag, fourth-order Butterworth filter with a cut-off frequency at 7 Hz.

#### Data analysis

Kinematic data and rectified EMG signals were superposed to determine the total trunk angle corresponding to EMG cessation during the flexion phase and the total trunk angle of EMG onset during the extension phase. EMG cessation and onset were quantified by visual inspection of the rectified EMG signal. The normalized root mean square (RMS calculated using a 250 ms moving window) value (normalized to the RMS value in extension during the first trial) during each phase of movement was calculated. The EMG data obtained from the left and right sides were averaged for L2 and L5 (no statistical difference were noted on each movement phase). Dependent variables included: (1) average total trunk flexion angle corresponding to the onset and cessation of myoelectric silence of the FRP and (2) average normalized EMG amplitude signals (RMS) during the full flexion phase of movement.

Two adjacent LEDs were used to create a vector, and the angles between vectors served to quantify knee, lumbar spine and pelvic motion. The knee angle was calculated by the angle between the lateral malleolus - head of the fibula and the femur’s lateral condyle - greater trochanter. Pelvic motion was determined by the angle between the ASIS-PSIS and greater trochanter-knee vectors, whereas lumbar spine motion was obtained by the angle between the T11-L1 and ASIS-PSIS vectors. Total trunk flexion angle was calculated as the sum of the lumbar spine and hip angles. Total flexion and extension angles were divided into quartiles (Q1-Q4) for which lumbar and hip movements were calculated.

#### Statistical analysis

The total flexion angle corresponding to the onset and cessation of myoelectric silence, the normalized EMG values during the full flexion phase of movement as well as all kinematic data were compared across the different experimental conditions using a 2 × 2 (intervention × load) repeated-measures analysis of variance (ANOVA). Spearman’s correlation coefficient was used to assess the correlation between changes in the various neuromechanical variables and reported changes in clinical outcomes. For all analyses, statistical significance was set at *p* < 0.05.

## Results

### Clinical outcomes

All participants had a history of CLBP for more than 24 months. Participants’ demographics as well as baseline clinical outcomes are respectively reported in Table 2 and Table 3. The statistical analyses revealed significant decreases in the mean ODI and FABQ II scores (physical activity) following surgery (dependent sample *t*-test; *p* < 0.05). The improvement in these scores did not reach the suggested clinically important difference (CID), with differences of 12.4 points for ODI (CID: 12.8 points) and 5.8 points for FABQ II (CID: 9 points) [43,44]. The 1.4 points decrease observed for the VAS scores indicates clinical improvement (clinically important difference (CID) of 1.20 to 1.74 point) but did not reach statistical difference [44,45]. No statistical difference or CID were noted for the FABQ I (work) (CID: 12 points) questionnaire (dependent sample *t*-test; *p* < 0.05) following surgery [43,44]. Nine and three participants respectively showed CID for VAS and FABQ I outcomes after the surgery, whereas two participants showed increased VAS scores and one participant an increased FABQ I score. Trunk flexibility, measured by the sit and reach test, did not change following surgery (dependent sample *t*-test; *p* > 0.05). No major surgical complication was reported, and all participants participated to both biomechanical laboratory testing. Noticeably, twelve participants out of fifteen had two or more surgical interventions, such as an anterior fusion, in combination with the prosthesis.

**Table 2:** Participants’ characteristics

**Characteristics**	**Means ± SDs**
Age (years)	43.5 ± 8.6
Weight (kg)	75.5 ± 10.1
Height (m)	1.74 ± 0.8
Body mass index (kg/m^2^)	25.0 ± 2.7
Level of disc prosthesis	
-L2-L3	n = 1
-L3-L4	n = 1
-L4-L5	n = 11
-L3-L4 and L4-L5	n = 1
-L5-S1	n = 1
Other surgery	
-fusion L5-S1	n = 12
Weeks before surgery*	12.1 ± 13
Weeks after surgery**	17.3 ± 8.4

Kg: kilograms.

M: meter.

SDs: standard deviations.

*Number of weeks between the initial laboratory experimentation and the surgery.

** Number of weeks between the surgery and the second laboratory experimentation.

**Table 3:** Outcome measures: VAS, ODI and FABQ I and II and flexibility

**Questionnaires**	**Before**	**After**	***p***
VAS ( /10)	4.8 ± 2	3.4 ± 2.8	0.06
ODI ( /100)	38.2 ± 13.7	25.8 ± 19.2	**0.03**
FABQ I ( /42)	22.1 ± 14.8	18 ± 15.2	0.09
FABQ II ( /24)	13.1 ± 9.1	7.3 ± 7.5	**0.01**
Sit and reach (cm)	8.2 ± 10.4	7.5 ± 12.7	0.74

Mean ± standard deviation for the different baseline characteristics.

FABQ I indicates fear-avoidance beliefs about work; FABQ II, fear avoidance beliefs about physical activity.

### Kinematics

**Figure 1:**
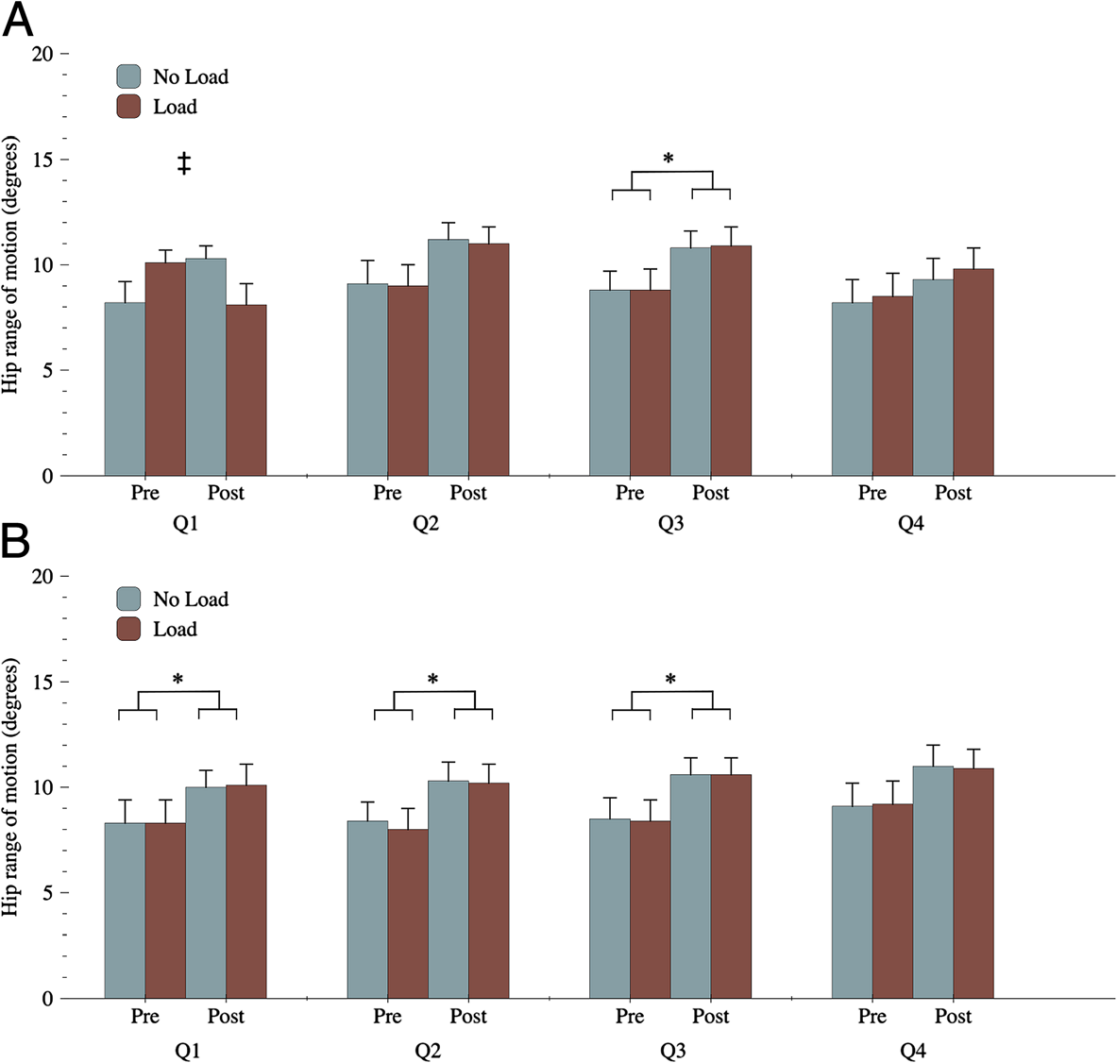
**Hip movement (°) during flexion (A) and extension (B) quartiles before and after surgery.** Increased hip ROM was observed after surgery for the following quartile (Q): extension Q1 (*p* = 0.03, η_p_^2^ = 0.34), extension Q2 (*p* = 0.03, η_p_^2^ = 0.35), extension Q3 (*p* = 0.04, η_p_^2^ = 0.29) and flexion Q3 (*p* = 0.04, η_p_^2^ = 0.30). * Treatment effect ρ < 0.05, and ‡ interaction effect ρ < 0.05.

The total trunk ROM in flexion significantly increased after the surgery (61.4° ± 23.1 vs. 69.8° ± 17.2; *p* = 0.02, η_p_^2^ = 0.34). A significant change in ROM was observed in the hip contribution to total flexion (33.6° ± 13.6 vs. 41.0° ± 10.6; *p* = 0.01, η_p_^2^ = 0.37) and was almost entirely responsible for the changes observed in explaining the total trunk ROM in flexion. Moreover, when all trials were considered (with and without a load), the changes in hip ROM observed after surgery were characterized by significant increases in movement during extension Q1, Q2 and Q3 and during flexion Q3. No change in lumbar ROM was observed following surgery (*p* = 0.73) and lumbar ROM was highly variable between participants. Kinematic data are reported in Table 4 (lumbar, hip and total trunk flexion) and Figure 1 (hip movement during flexion/extension quartiles), where absolute changes in hip ROM for each quartile of flexion and extension movements are presented. There were no differences in knee angle recorded before and after the surgical intervention.

**Table 4:** Kinematic data in flexion

**Flexion**	**Degree**	**CI 95%**	***p***
Total angle			
Pre	61.4 ± 23.1	48.6 – 74.3	**0.02**
Post	69.8 ± 17.2	60.2 – 79.3	
Hip total angle			
Pre	33.6 ± 13.6	26.0 – 41.1	**0.01**
Post	41.0 ± 10.6	35.1 – 46.9	
Lumbar total angle			
Pre	27.4 ± 11.4	21.1 – 33.7	0.73
Post	28.2 ± 9.6	22.8 – 33.5	

Mean ± standard deviation and 95% confidence interval (CI 95%) for the different angles.

### Electromyography

**Figure 2:**
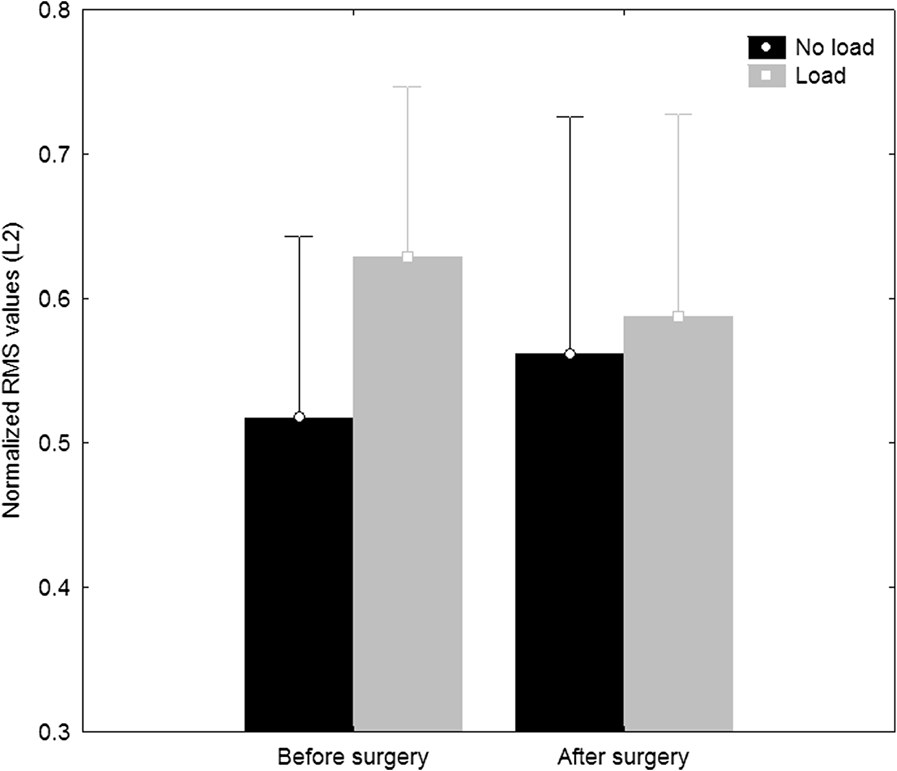
Pre- and post-surgery normalized root mean square (RMS) values of ES at L2 during the flexion relaxation phase (FRP) of movement.

Loading the spine significantly increased (*p* < 0.05) RMS values of the ES muscles at L2 through all phases of the flexion-extension task (quiet standing, flexion, extension and FRP). Under loading condition, the ES RMS values at L5 increased for the extension (*p* = 0.03) and FRP phases only (*p* = 0.02). A main effect of surgery was noted for ES RMS values at L2 during the quiet standing position (0.40 ± 0.04 vs. 0.33 ± 0.05; *p* < 0.05, η_p_^2^ = 0.36) and at L5 during the flexion phase for which ES RMS values significantly increased following surgery (0.79 ± 0.05 vs. 0.91 ± 0.03; *p* < 0.05, η_p_^2^ = 0.35). As illustrated in Figure 2, the ANOVA also yielded significant surgery x load interaction effects for ES RMS values at L2 during the FRP phase ( *p* = 0.008, η_p_^2^ = 0.40). No significant surgery x load interaction could be observed at L5 for ES RMS values.

**Figure 3:**
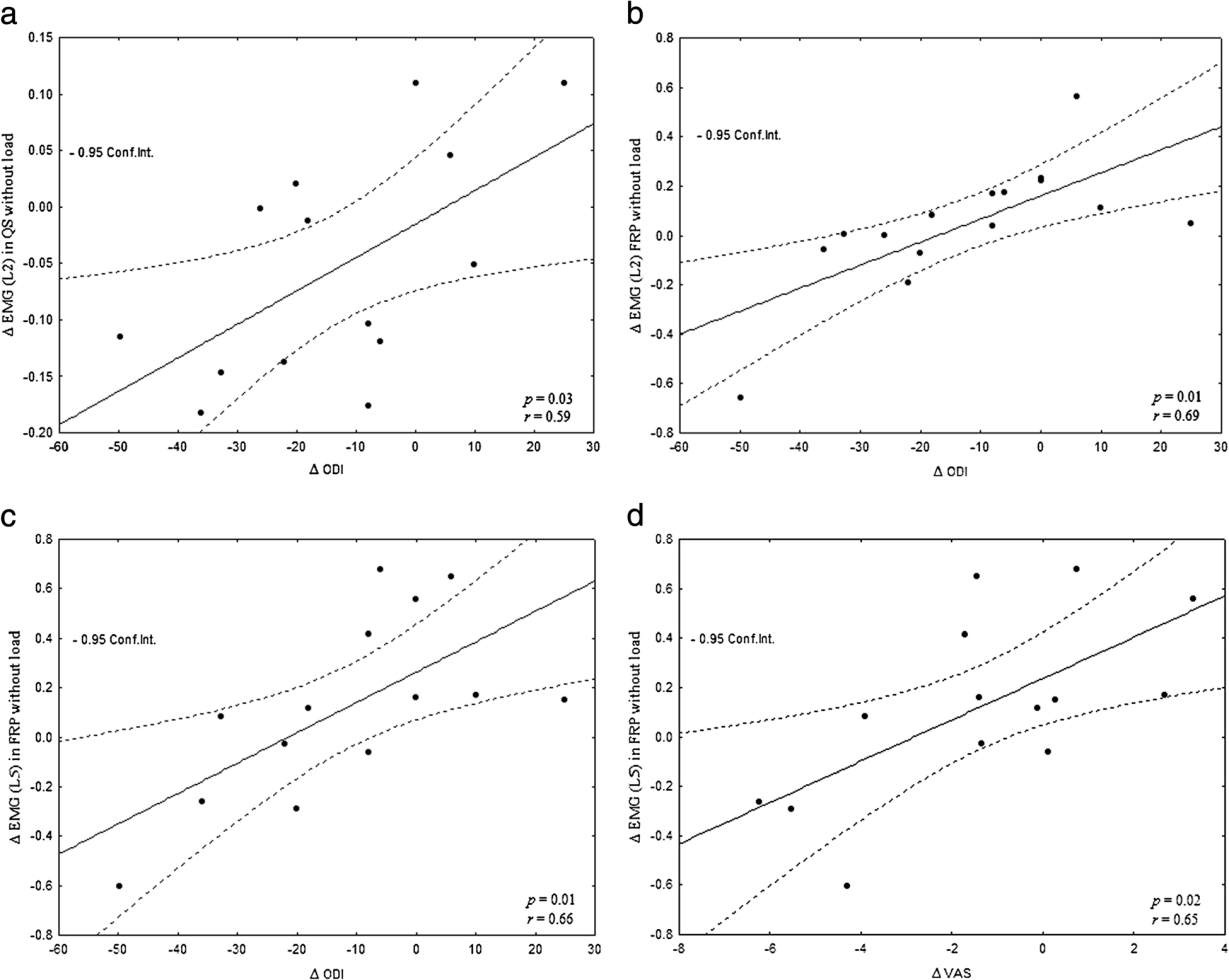
**Significant correlations between changes in EMG values (Δ EMG) at the ES at L2 (a and b) and L5 (c and d) and reported changes in clinical outcomes.** Flexion relaxation phase (FRP), quiet standing (QS) changes in Oswestry Disability Index (∆ ODI) and visual analogue scale (∆VAS), ρ: statistical significance and *r*: correlation.

No statistically significant correlation was found between clinical outcomes (pain, disability, fear- avoidance questionnaires) and kinematic variables. On the other hand, positive correlations were observed between changes in ES RMS values during the FRP phase and several clinical outcome measures. In fact, changes in Oswestry scores were positively and strongly correlated to changes in ES RMS at L2 (both with and without load: *r* = 0.69, *p* = 0.01) and L5 (with load: *r* = 0.71, *p* = 0.01; without load: *r* = 0.67, *p* = 0.01). During the FRP phase, the FABQ I questionnaire was positively and strongly correlated to changes at L2 without load (*r* = 0.56, *p* = 0.04) and at L5 under the loading condition (*r* = 0.59, *p* = 0.04), whereas changes in VAS scores were positively and strongly correlated to changes in ES RMS at L5 (with load: *r* = 0.72, *p* = 0.01; without load: *r* = 0.65, *p* = 0.02). ODI questionnaire was also strongly correlated to changes in ES contraction at L2 during the quiet standing position (with load: *r* = 0.72, *p* = 0.00; without load: *r* = 0.59, *p* = 0.03). Only one negative correlation was found between the FABQ II questionnaire and changes in ES RMS at L2 during flexion (without load: *r* = −0.59, *p* = 0.03). Significant correlations between changes in EMG values (without load) and reported changes in clinical outcomes (ODI and VAS) are illustrated in Figure 3.

## Discussion

The main objective of this study was to assess whether or not lumbo-pelvic neuromechanical adaptations occurred following lumbar disc replacement surgeries, and if such changes were related to changes in clinical outcomes. Almost four months following the disc replacement procedure, participants reported a significant reduction in their disability scores and fear-related beliefs for physical activities. These subjective findings in clinical outcomes were coupled with an increase in pelvic flexion as well as a reduction in ES muscle activity in quiet standing, while muscle activity increased during the flexion phases at L5 following surgery. Interestingly, decreases in EMG activity in superficial lumbar spine muscles during the FRP were correlated to decreases in pain, disability and fear-avoidance beliefs scores, whereas changes in lumbar and hip flexion were not correlated to clinical outcomes.

### Clinical outcomes

The present study’s small sample size and its design preclude from broad clinical outcome comparisons with other studies. However, arthroplasty and arthrodesis comparative studies have demonstrated significant improvement of ODI and VAS scores at three months follow-up, which is similar to this study for the ODI only [9-11]. FABQ has not been used in any lower back surgery trials.

### Kinematic

Arthroplasty clinical trials have mainly focused on the implant’s efficacy, whereas their biomechanical properties have been less frequently described. The ROM comparisons before and after surgery (in vivo) are generally done by radiographic analysis only. When segmental movement was assessed using plain film radiographs following surgery, the flexion-extension motion was found either preserved or superior to the presurgical status [10,11,18-20] McAffe et al. [19] showed a decrease of the radiographic ROM at the three-month follow-up compared to ROM before surgery. The authors also reported continuous improvement in ROM at 6, 12 and 24 months. Given the results of the present study, it is therefore possible that initial adaptations following surgery are mostly aimed at reducing mechanical stresses on the operated segments (consequently increasing hip movement to maintain functional capacity). In a study comparing segmental contribution to total ROM following disc replacement or fusion (two-year follow-up), Auerbach et al.[20] found that the total lumbar ROM was increased in patients who received a disc replacement at L4-L5. However, the ROM of patients receiving disc replacement at L5-S1 was similar to those of patients who underwent a fusion surgery at the same level [20]. When considering the surgical technical accuracy, McAfee et al. [19] showed that flexion-extension motion improved with proper alignment of the prosthesis, whereas other authors did not find any correlation between the positioning of disc prosthesis and clinical outcomes (VAS and ODI) [46]. Several limitations, however, should be considered as potential sources of error measurement [20,47] when ROM is assessed using plain film radiographs, such as examiner errors, non-uniformity of the vertebral endplates, film quality, subject’s positioning and subject’s effort-related variability during flexion-extension image acquisition. In the current study, participants showed an increase in trunk ROM that was mainly explained by increased the hip’s contribution to movement. It is therefore difficult to link the ROM improvement to the procedure, and such drastic changes in hip ROM may represent an alternative motor pattern aimed at limiting movement and loading of lumbar segments. Long term assessments of movement strategies are needed to explore this hypothesis.

### Electromyography

As described by Gupta [29] as well as Descarreaux et al. [48], in healthy subjects, the loading condition led to an increase of the RMS values at L2 during all the phases of the flexion-extension task, reflecting the need for additional muscular contraction to counteract the increased flexion moment generated by the load. However, this phenomenon was observed only during the extension and the FRP phases at L5. On the other hand, Sarti et al. [31] have not found any effect of additional loading of the trunk on the FRP. While no change in lumbar spine kinematics could be observed during movement, one interesting finding related to the possible surgical effect was the increased muscular activity at L5 in the flexion phase. Together with the increased hip ROM, the absence of any significant RMS changes at L5 during other movement phases may support the hypothesis that an alternative motor pattern for trunk flexion was established by the participants. One interesting study, which assessed the FRP before and after lumbar discectomy (four weeks), showed that neither FRP nor ROM significantly improved after surgery despite significant clinical improvement [37]. Conversely, our study showed, that under challenging conditions (loading the spine), participants were able to stabilize the lumbar using less erector spinae activation. Short term follow-up evaluations (four weeks in the Wallbom et al. [37] study and 17 weeks in the present study) may not be representative of the overall benefit of spinal surgery. Tissue recovery may not be completely obtained, and other psychosocial factors such as fear-avoidance beliefs may temporarily limit functional capacities. Long term follow-up evaluations are needed to better understand neuromechanical changes and the potential role of rehabilitation following lower back surgery.

### Clinical interpretation

Following surgery, the participants did not attend a specific rehabilitation program, physiotherapy or any other conservative treatment regimen. The general indications given by the surgeon were to continue the usual daily life activities as much as possible, and not perform trunk flexion during the first six weeks. Other studies have shown clinical improvement (physical and/or disability) with aggressive rehabilitation after a lumbar discectomy when compared to less active training programs [49-51]. One study showed statistically significant improvement in patients’ clinical status (VAS and ODI) after early rehabilitation program designed for patients with disc replacement surgery [52]. The program included dynamic lumbar stabilization exercises and kinetic-chain strengthening. In this study, patients had only one level of surgical intervention, and no control group to compare the clinical results to. With a complete rehabilitation program, Neblett et al. [32] studied the functional restoration of abnormal flexion-relaxation response with CLBP participants. The rehabilitation program included a surface EMG-assisted stretching biofeedback training protocol, physical exercise (daily group and individual stretch training) and cognitive-behavioral counselling (educational classes, relaxation, biofeedback, stress management training and multimodal disability management) over two months. The final evaluation demonstrated that 86% of the rehabilitation group achieved flexion-relaxation, compared to 34% at the initial stage, and were similar to the pain-free control group. Interestingly, 31% of the participants in the rehabilitation group had a spinal fusion; however, the authors did not specify if the FRP was present before and after the rehabilitation program for this specific group. Future studies should evaluate long term changes in FRP parameters following disc replacement surgery, with and without long term rehabilitation.

As suggested by several authors, the abnormal FRP can result from an initial fear-avoidance reaction to back pain triggering and over time evolve into an unintentional protection mechanism [28,32,53]. Interestingly, the correlations between the clinical outcomes (VAS, ODI and FABQ I) and the biomechanical changes during the FRP in the present study seem to suggest that the biomechanical changes observed following surgery are closely related to changes in pain perception, fear avoidance beliefs and physical disability.

### Limitations

One important limitation of this study is related to the surgical interventions. Twelve participants out of 15 had two or more surgical interventions with the disc prosthesis. The additional interventions were an anterior vertebral fusion performed with metal implants and/or an implantation of an interbody cage below the prosthesis level. It is therefore possible that any potential gain obtained with a disc replacement surgery could have been invalidated when fusion was performed. According to a recent review, this seems to reflect not only clinical practice but also how current low back surgical studies are designed [54]. The result of the present study may reflect those obtained in standard orthopedic care where patients with DDD often present with multiple spinal conditions. They must, however, be interpreted with caution as broad generalization is not possible since the sample size was relatively small and there was no control group involved. However, the changes observed in the present study (both in clinical and neuromechanical outcomes) showed large effect size and often reached clinical significance.

## Conclusion

This study assessed the neuromechanical adaptations following a lumbar disc replacement surgery. Surface-EMG, kinematic and questionnaires (pain, disability and fear avoidance-related beliefs) were used to evaluate participants’ functional and clinical status. Improvement in disability and fear avoidance beliefs linked to physical activities were observed following surgery. Although lumbar ROM did not change after disc replacement, a significant increase in the hip ROM during the flexion-extension task as well as changes in ES muscle activity was observed. Further research is needed to assess long term neuromechanical changes and the potential role of rehabilitation following lower back surgery.

## Competing interests

Statement of financial disclosure and conflict of interest: the authors declare that they have no competing interests.

## Authors’ contributions

MD contributed to the study design and protocol development, had overall responsibility for the conduct of the study, and contributed to the experimentation, data analysis, writing of the manuscript and supervision of JO. JO, as part of her master’s degree thesis, conducted all experimental sessions, statistical analysis, and manuscript preparation. JFR was responsible for all clinical intervention and overall patient management. All authors read and approved the final manuscript.
